# Prevalence of Brain MRI Markers of Hemorrhagic Risk in Patients with Stroke and Atrial Fibrillation

**DOI:** 10.3389/fneur.2016.00151

**Published:** 2016-09-20

**Authors:** Christopher Karayiannis, Cathy Soufan, Ronil V. Chandra, Thanh G. Phan, Kitty Wong, Shaloo Singhal, Lee-Anne Slater, John Ly, Chris Moran, Velandai Srikanth

**Affiliations:** ^1^Department of Medicine, Stroke and Ageing Research Centre, School of Clinical Sciences at Monash Health, Monash University, Melbourne, VIC, Australia; ^2^Neuroradiology Service, Monash Imaging, Monash Health, Melbourne, VIC, Australia; ^3^Stroke Unit, Neurosciences, Monash Health, Melbourne, VIC, Australia; ^4^Department of Medicine, Peninsula Health, Melbourne, VIC, Australia; ^5^Central Clinical School, Monash University, Melbourne, VIC, Australia; ^6^Aged Care, Alfred Health, Melbourne, VIC, Australia

**Keywords:** atrial fibrillation, stroke, microbleeds, MRI

## Abstract

**Background and purpose:**

Cerebral microbleeds (CMBs), cortical superficial siderosis, white matter lesions (WML), and cerebral atrophy may signify greater bleeding risk particularly in patients in whom anticoagulation is to be considered. We investigated their prevalence and associations with stroke type in patients with stroke and atrial fibrillation (AF).

**Materials and Methods:**

Cross-sectional sample, Monash Medical Centre (Melbourne, Australia) between 2010 and 2013, with brain MRI. MRI abnormalities were rated using standardized methods. Logistic regression was used to study associations adjusting for age and sex.

**Results:**

There were 170 patients, mean age 78 years (SD 9.8), 154 (90.6%) with ischemic stroke. Prevalence of MRI markers were any microbleed 49%, multiple (≥2) microbleeds 30%, confluent WMLs 18.8%, siderosis 8.9%, severe cerebral atrophy 37.7%. Combinations of the severe manifestations of these markers were much less prevalent (2.9–12.4%). Compared with ischemic stroke, those with hemorrhagic stroke were more likely to have ≥10 microbleeds (OR 5.50 95% CI 1.46–20.77, *p* = 0.012) and siderosis (OR 6.24, 95% CI 1.74–22.40, *p* = 0.005). Siderosis was associated with multiple microbleeds (OR 8.14, 95% CI 2.38–27.86, *p* = 0.001). Patients admitted with hemorrhagic stroke and multiple microbleeds were more frequently anticoagulated prior to stroke (6/7, 85.7%) than in those with single (1/2, 50%) or no microbleeds (4/7, 57%).

**Conclusion:**

Multiple CMBs, severe WML, and severe cerebral atrophy were common individually in hospitalized patients with stroke and AF, but less so in combination. A higher burden of CMBs may be associated with intracerebral hemorrhage in stroke patients with AF.

## Introduction

**Figure 1:**
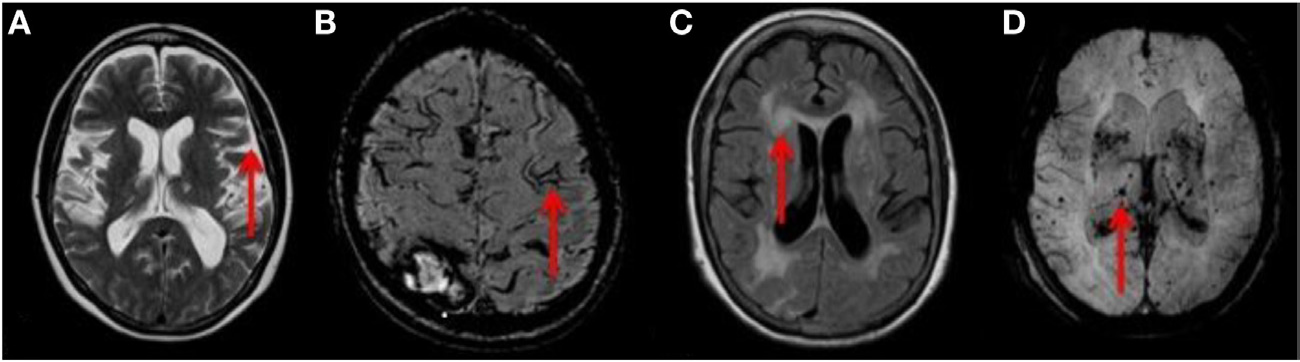
**(A)** T2 weighted axial MRI of the brain showing severe cerebral atrophy [Grade 4 rating using a published reference template (31)] with sulcal widening (red arrow). **(B)** Axial MRI of the brain demonstrating a linear gyriform hypointensity (red arrow) on SWI sequence in a cortical distribution representative of superficial siderosis. **(C)** T2 FLAIR weighted MRI showing large confluent lesions (red arrow) with a Fazekas 3 rating as described in the Section “Materials and Methods.” **(D)** SWI MIP sequence MRI of the brain showing multiple diffuse microbleeds (red arrow). Microbleeds were assessed using the validated Brain Observer Microbleed Scale (BOMBs) criteria as discussed in the Section “Materials and Methods.”

Atrial fibrillation (AF) is a common and important risk factor for stroke in older people (1). Anticoagulation is a highly effective therapy for secondary prevention in patients with AF and previous ischemic stroke (2), but many such patients are not treated for fear of intracerebral hemorrhage (ICH) (3). Although there are clinical tools that can be used to predict overall bleeding risk (4), they do not incorporate the presence of relevant brain lesions that may heighten risk. Abnormalities on brain MRI, such as cerebral microbleeds (CMBs), cortical superficial siderosis (cSS), confluent white matter lesions (WML), and cerebral atrophy (Figure 1), are commonly seen in older people in the general population, attending memory clinics, and with stroke (5–8). A greater burden of such abnormalities may signify a greater future risk of ICH, when considering anticoagulation, although small sample sizes and suboptimal study designs have left uncertainty regarding this issue (9–12).

Cerebral microbleeds are small hypointense lesions visible on susceptibility weighted imaging (SWI) or gradient echo (GRE) MRI sequences, representing hemosiderin deposits (13). Multiple lobar CMBs are associated with cerebral amyloid angiopathy, while infratentorial or deep CMBs are associated with cardiovascular risk factors, such as hypertension (8, 14). CMBs are associated with an increased risk of hematoma expansion in patients with ICH (15) and may also increase the risk of ICH in patients receiving antiplatelet drugs (16, 17) or thrombolysis (18). CSS is caused by repeated clinical or subclinical bleeding into the cortical subarachnoid space leading to hemosiderin deposition (13) and is visible on SWI or GRE sequences (Figure 1). It is associated with cerebral amyloid angiopathy (19) and an increased risk of ICH (9). Age-related WML are ubiquitous in older people, are associated with a history of hypertension (20), and are closely associated with the presence of CMBs in adults aged over 40 years (21). A large burden of WML (confluent WML) is associated with physical and cognitive frailty (22), predicting mortality after ICH (23) and spontaneous ICH (24). WML are best identified as hyperintensities on FLAIR sequences as hyperintense lesions (25). Cerebral atrophy is associated with cognitive impairment in patients with stroke (26, 27), may indicate cognitive frailty, and thus be a proxy marker for unfavorable outcome after spontaneous ICH (11).

Although there are published data on the prevalence of each of these MRI markers in isolation, it is unclear how commonly they coexist in patients with stroke and AF. We sought to evaluate the prevalence of these MRI abnormalities in a hospital-based sample with stroke and AF, their relationships with each other, and with stroke type (hemorrhagic versus ischemic).

## Materials and Methods

### Sample Ascertainment

This was a cross-sectional study with retrospective ascertainment of data. The sample consisted of patients with stroke admitted to the Stroke Unit at the Monash Medical Centre, Melbourne, Australia between October 2010 and October 2013. The hospital electronic record system was used to identify patients 18 years or older, with confirmed AF diagnosed based on ECG, Holter monitoring, or documented medical diagnosis, and with MRI performed as part of their diagnostic work-up within 4 months of the index stroke event. Ethics approval was granted by the Monash University Health and Medical Research Ethics Committee and the Monash Health Research Directorate. Individual informed consent was not required because the project was deemed low-risk given the retrospective design, use of routinely collected medical information, and the de-identified reporting of aggregated results.

### MRI Measures

A fellowship trained neuroradiologist with 6 years of experience in MR imaging, who was blinded to clinical details, rated all MRI scans for acute or previous stroke, CMBs, cSS, WML, and cerebral atrophy. CMBs were assessed on SWI sequence and then rated using the validated Brain Observer Microbleed Scale (BOMBs) criteria (28). The BOMBs scale provides a measure of the number of microbleeds, the size of each (<5 or 5–10 mm), and the location within the brain and brainstem (28). We defined cSS as a linear gyriform hypointensity on SWI sequence in a cortical distribution without corresponding hyperintense signal on FLAIR or T1 weighted sequences (7, 29). FLAIR sequences were reviewed to identify and rate WML using the Fazekas scale as mild, moderate, or severe (confluent) lesions (30). Cerebral atrophy was rated using a published reference template (31), and severe atrophy was defined as >75th percentile for that patient’s age-group based on the template.

### Other Measures

Additional information gathered included age, sex, date of birth, cardiovascular risk factors, the use of anticoagulation therapy, or antiplatelet therapy prior to stroke and international normalized ratio (INR) level if applicable.

### Statistical Analysis

Prevalence proportions of each type of MRI abnormality and combinations of MRI abnormalities were estimated. Chi-square and *t*-tests were used for comparison of categorical or continuous variables. Logistic regression was used to study associations of MRI abnormalities with each other and with ICH (compared with ischemic stroke), adjusting for age and sex.

## Results

**Figure 2:**
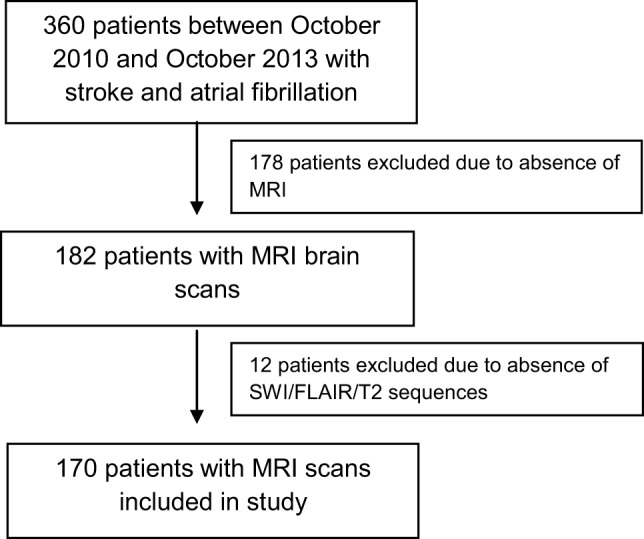
**Flow diagram of sample ascertainment**.

During the study period, a total of 360 patients with stroke and AF were identified. Of these, 178 were excluded due to MRI not being performed. This was either due to death before 4 months or because institutional protocols did not demand compulsory acute or follow-up MRI in all patients, with CT brain and perfusion imaging being the preferred acute imaging modality. A further 12 patients were excluded because SWI sequence was not available or was degraded, leaving 170 patients included in the final analysis (Figure 2). The mean age of the sample was 78 years (SD 9.8), and 53% were males. There were 154 (90.6%) patients with ischemic strokes and 16 (9.4%) with ICH (Table 1). The median delay from stroke event to MRI scan was 13 days. Patients who did not undergo MRI were older on average, with a higher 90-day modified Rankin Scale score and CHADS_2_ score, and were more likely to have a diagnosis of ischemic heart disease (Table 1) but were similar with respect to sex, other cardiovascular risk factors, stroke type, antiplatelet, and anticoagulant use.

**Table 1:** **Sample characteristics – all figures reported are *n* (%), unless otherwise specified**.

Variable	Included (*n* = 170)	Excluded^a^ (*n* = 190)	*p* value
Mean age	77.7 years (SD 9.81)	80.9 years (SD 9.93)	0.0022
Female sex	80 (47.0)	99 (52.1)	0.34
Current smoker	64 (37.7)	54 (28.4)	0.063
Hypertension	157 (92.4)	173 (91.0)	0.66
Dyslipidemia	116 (68.2)	125 (67.8)	0.62
Ischemic heart disease	89 (52.4)	119 (62.6)	0.049
Diabetes mellitus	45 (26.5)	49 (25.8)	0.88
Pre-admission antiplatelet therapy	95 (55.9)	97 (51.0)	0.36
Pre-admission anticoagulation therapy	57 (33.5)	70 (36.8)	0.51
Pre-admission combined therapy	6 (3.5)	8 (4.2)	0.74
Ischemic stroke	154 (90.6)	163 (85.8)	0.11
Hemorrhagic stroke	16 (9.4)	27 (14.2)	
Systolic BP ≥160 mmHg^b^	92 (54.1)	94 (49.5)	0.26
CHADS_2_	3.1 points (SD 1.3)	3.4 points (SD 1.3)	0.016
90-day modified Rankin Scale ≥3	65 (38.2)	138 (72.6)	<0.001

*^a^Excluded due to lack of MRI or failed/inadequate MRI*.

*^b^Systolic BP ≥160 mmHg on admission*.

Prevalence proportions of individual MRI markers in the sample were as follows: any CMB 49% (lobar distribution in 94% of patients with CMB), multiple (≥2) CMBs 30%, confluent WML 18.8%, cSS 8.9%, and severe atrophy 37.7%. The proportions of patients who had CMBs together with other markers ranged from 4.1 to 20.0% (Table 2), depending on the combination. Among patients with ischemic stroke, 6.5% (*n* = 10) were found to have cSS, only one of whom had a known clinical history of ICH. The proportions of patients with coexistent severe manifestations of the markers were relatively low (2.9–12.4%) (Table 2). Adjusting for age and sex, the presence of siderosis was strongly associated with multiple CMBs (OR 4.85, 95% CI 1.15–20.45, *p* = 0.031). Of the 11 patients with both multiple CMBs and cSS, all had lobar CMBs, while only 4 had deep CMBs.

**Table 2:** **Prevalence of MRI abnormalities in sample**.

MRI marker	All (*n* = 170)	Ischemic stroke (*n* = 154)	ICH (*n* = 16)
	
In isolation		*N* (%)	
Any CMB	83 (49.0)	74 (48.0)	9 (56.3)
Multiple CMB (≥2, mCMB)	51 (30.0)	44 (28.6)	7 (43.8)
cSS	15 (8.9)	10 (6.5)	5 (31.3)
Confluent WML	32 (18.8)	29 (18.8)	3 (18.8)
Severe atrophy	64 (37.7)	59 (38.3)	5 (31.3)
CMB + cSS	11 (6.5)	7 (4.5)	4 (25.0)
mCMB + cSS	11 (6.5)	7 (4.5)	4 (25.0)
CMB + severe atrophy	34 (20.0)	30 (19.5)	4 (25.0)
mCMB + severe atrophy	21 (12.4)	18 (11.7)	3 (18.8)
CMB + confluent WML	21 (12.4)	19 (12.3)	2 (12.5)
mCMB + confluent WML	15 (8.8)	13 (8.4)	2 (12.5)
Confluent WML + severe atrophy	14 (8.2)	14 (9.1)	0 (0.0)
CMB + confluent WML + severe atrophy	7 (4.1)	7 (4.5)	0 (0.0)
mCMB + confluent WML + severe atrophy	5 (2.9)	5 (3.2)	0 (0.0)
CMB + confluent WML + severe atrophy + cSS	0 (0.0)	0 (0.0)	0 (0.0)

*CMB, cerebral microbleed; mCMB, multiple cerebral microbleeds (≥2 microbleeds); cSS, cortical superficial siderosis; WML, white matter lesions*.

**Figure 3:**
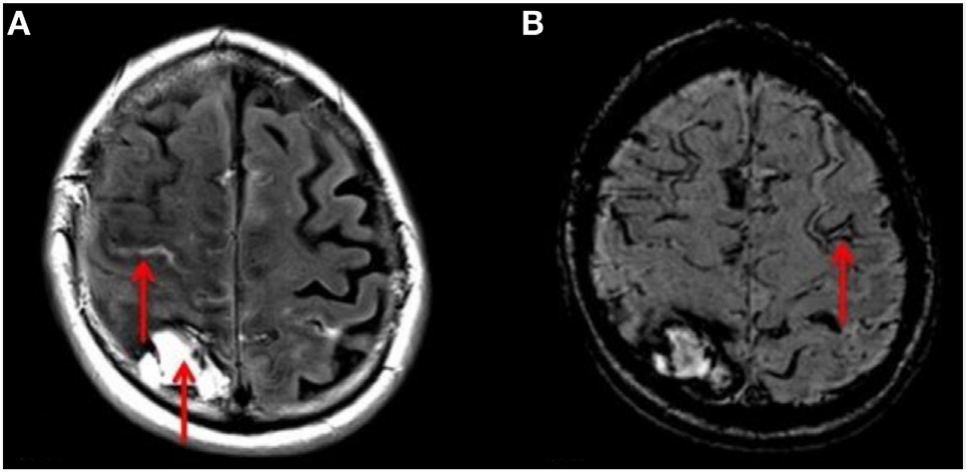
**(A)** T2 FLAIR axial MRI of the brain showing right-sided ICH and acute bleeding (red arrows). **(B)** SWI sequence MRI of the same patient demonstrating a linear gyriform hypointensity in a cortical distribution (red arrow), representative of siderosis, in a region independent to that of the site of hemorrhage.

In unadjusted comparisons between those with ischemic stroke and ICH (Table 3), the proportions of patients with any CMBs, multiple CMBs, confluent WML, and severe atrophy were similar between groups (*p* > 0.05), whereas cSS was more prevalent in those with ICH (*p* = 0.001). Adjusting for age and sex, there was no significant association between ICH and the presence of CMB (OR 1.29, 95% CI 0.44–3.75, *p* = 0.64). But the strength of the association between ICH and CMB increased with the burden of CMBs with OR 1.80 (95% CI 0.62–5.83, *p* = 0.28) in those with ≥2 CMBs, OR 2.82 (95% CI 0.80–9.93, *p* = 0.107) in those with ≥5 CMBs, and OR 5.50 (95% CI 1.46–20.77 *p* = 0.012) in those with ≥10 CMBs. ICH was significantly associated with the presence of cSS (OR 6.24, 95% CI 1.74–22.40, *p* = 0.005). In all cases of cSS within patients suffering ICH, cSS was not only present in relation to the location of the index clinical bleed but also in areas distant to the index bleed and in the contralateral hemisphere (Figure 3). ICH was not associated with either the presence of confluent WML (OR 0.95, 95% CI 0.24–3.73 *p* = 0.95) or severe cerebral atrophy (OR 0.62, 95% CI 0.19–2.03, *p* = 0.43).

**Table 3:** **Association of MRI markers with stroke type**.

MRI marker	Odds ratio for ICH (95% CI)	*p* value
CMB ≥2	1.80 (0.62, 5.83)	0.28
CMB ≥5	2.82 (0.80, 9.93)	0.11
CMB ≥10	5.50 (1.46, 20.77)	0.012
cSS	6.24 (1.74, 22.40)	0.005
Confluent WML	0.95 (0.24, 3.73)	0.95
Severe atrophy	0.62 (0.19, 2.03)	0.43
CMB ≥2 and cSS	4.85 (1.15, 20.45)	0.031

In the whole sample, 57/170 (33.5%) patients were anticoagulated prior to stroke, more so in those presenting with ICH (11/16, 68.8%) than in those with ischemic stroke (47/154, 30.5%, *p* = 0.002). On the other hand, 95/170 (55.9%) received antiplatelet therapy prior to stroke, more so in those presenting with ischemic stroke (93/154, 60.4%) than in those with ICH (2/16, 12.5%, *p* < 0.001). Of the five patients with ICH and cSS, three were on anticoagulants prior to stroke with INRs ranging from 2.9 to 3.7, and none were on antiplatelet therapy. Among patients with ICH, prior anticoagulant use was more common in those with mCMBs (6/7, 85.7%) than in those with single (1/2, 50%) or no CMB (4/7, 57%), but these comparisons did not reach statistical significance (*p* = 0.3).

## Discussion

In this study, we present data on the prevalence of several MRI markers that may predict risk of future ICH in patients with stroke and AF. We found that CMBs, severe WML, and cerebral atrophy were each highly prevalent in this sample, whereas the prevalence proportions for cSS and for combinations of markers were less common. The presence of cSS was strongly associated with the presence of multiple CMBs. In our study, patients with AF and ICH more often had cSS and a higher burden of CMBs than those with AF and ischemic stroke.

Our data are unique in providing prevalence estimates of MRI markers of vascular brain disease individually and together in a sample of patients with stroke and AF. Previous studies have reported on the frequency of such MRI markers individually and in various types of samples, including patients with stroke. In a recent comprehensive review, it was reported that the prevalence of CMBs ranged from ~18 to ~68% in patients with ischemic stroke (32). Our estimates (~50%) fall broadly within this range. Prevalence estimates of cSS in this patient group have not been reported previously. In our sample, cSS was more prevalent in patients admitted for ICH, strongly related to the presence of multiple CMBs, consistent with studies of the general population and memory clinic patients (7, 33). We also found that cSS was present at sites both related and unrelated to the index bleed. Therefore, it is possible that patients with stroke and AF who have imaging evidence of cSS may be at high risk of bleeding from anticoagulation therapy, and this needs further clarification.

Patients with a history of ischemic stroke and AF are at high risk for developing recurrent stroke. There is no consensus currently on what approach should be taken in such patients who have cSS and mCMBs on MRI, but data from larger samples may contribute to risk stratification algorithms that have been proposed (34). In our sample, ~7% of patients with ischemic stroke and AF also showed evidence of prior cSS, despite an absence of history of ICH in all but one patient. In one series of patients with cSS and probable cerebral amyloid angiopathy, as many as 50% of patients went on to develop future ICH within 3 years (35).

The combination of multiple CMBs and cSS may signify a particularly high risk of ICH. In our sample, ~20% of patients with ICH had both cSS and multiple CMBs, suggesting the presence of cerebral amyloid angiopathy (19). All such patients had lobar CMBs, supporting this contention. There is emerging evidence that patients with ischemic stroke and the presence of CMBs who undergo thrombolysis may be at ~2-fold increased risk of ICH (36). Our data, although cross-sectional, provide some evidence to suggest that an increasing burden of CMBs may be related to the risk of ICH, and the presence of a threshold effect may be worth exploring in prospective studies of patients with stroke and AF requiring anticoagulation. Confluent WML and severe cortical atrophy were relatively common in keeping with such an older sample with cerebrovascular disease. Although their prevalence was not markedly different between patients with ischemic stroke and ICH, it would still be of interest to see whether they predict risk of future ICH independent of CMBs, or whether the cumulative presence of all such markers (although relatively uncommon) indicates a greater risk of ICH in the setting of anticoagulation.

The main strength of this study is that it examines a clinically relevant high-risk sample for cardioembolic stroke. Additionally, the radiological ratings were performed blinded to clinical details. There were certain limitations. The sample was relatively small, and there were few patients with ICH, compared to ischemic stroke. Therefore, study results are more generalizable to patients with ischemic stroke and AF, than to those with ICH and AF. Furthermore, the retrospective and cross-sectional design did not allow an estimation of future risk of ICH in patients. The lack of MRI in some eligible patients renders our prevalence estimates open to the effect of selection bias, and it is likely that we underestimated prevalence given the older age of the excluded sample. Given the retrospective nature of the study, we did not have MRI routinely at stroke onset or prior to stroke, but a median of 13 days after stroke. This is unlikely to affect estimates of the MRI markers, which are usually chronic lesions. Furthermore, many of our patients were anticoagulated prior to stroke event. Given the cross-sectional and retrospective nature of data collection, it is possible that some CMBs may have developed after commencement of anticoagulation. Therefore, we cannot draw strong conclusions about the predictive utility of CMBs by themselves for the risk of ICH in patients with stroke and AF independent of anticoagulation. This issue can only be confirmed in prospective studies of patients who are not on anticoagulant therapy.

## Conclusion

Our study suggests that multiple CMBs, severe WML, and severe cerebral atrophy were common individually in hospitalized patients with stroke and AF, but less so in combination. There were differences in prevalence of these imaging markers between ischemic and hemorrhagic stroke, with patients with ICH more often having multiple CMBs and cSS.

## Author Contributions

CK, CS, TP, RC, and VS contributed to the conception and design of the study, the acquisition of data, and the analysis and interpretation of data. CK drafted the article, and CS, TP, RC, and VS revised it critically for important intellectual content. KW and SS were involved in acquiring and analyzing the data, and they revised the article critically for important intellectual content. LAS, JL, and CM were involved in the conception and design, as well as analysis and interpretation of data, and revising the article for important intellectual content. All authors gave final approval of the version submitted for publication and agreed to be accountable for all aspects of the work.

## Conflict of Interest Statement

The authors declare that the research was conducted in the absence of any commercial or financial relationships that could be construed as a potential conflict of interest.

## Abbreviations

AF, atrial fibrillation; CMB, cerebral microbleed; cSS, cortical superficial siderosis; ICH, intracerebral hemorrhage; WML, white matter lesions.
